# Machupo Virus Glycoprotein Determinants for Human Transferrin Receptor 1 Binding and Cell Entry

**DOI:** 10.1371/journal.pone.0021398

**Published:** 2011-07-07

**Authors:** Sheli R. Radoshitzky, Lindsay E. Longobardi, Jens H. Kuhn, Cary Retterer, Lian Dong, Jeremiah C. Clester, Krishna Kota, John Carra, Sina Bavari

**Affiliations:** 1 Toxicology Division, United States Army Medical Research Institute of Infectious Diseases (USAMRIID), Frederick, Maryland, United States of America; 2 Integrated Research Facility at Fort Detrick (IRF-Frederick), Division of Clinical Research (DCR), National Institutes of Allergy and Infectious Diseases (NIAID), National Institutes of Health (NIH), Fort Detrick, Frederick, Maryland, United States of America; The Scripps Research Institute, United States of America

## Abstract

Machupo virus (MACV) is a highly pathogenic New World arenavirus that causes hemorrhagic fever in humans. MACV, as well as other pathogenic New World arenaviruses, enter cells after their GP1 attachment glycoprotein binds to their cellular receptor, transferrin receptor 1 (TfR1). TfR1 residues essential for this interaction have been described, and a co-crystal of MACV GP1 bound to TfR1 suggests GP1 residues important for this association. We created MACV GP1 variants and tested their effect on TfR1 binding and virus entry to evaluate the functional significance of some of these and additional residues in human and simian cells. We found residues R111, D123, Y122, and F226 to be essential, D155, and P160 important, and D114, S116, D140, and K169 expendable for the GP1-TfR1 interaction and MACV entry. Several MACV GP1 residues that are critical for the interaction with TfR1 are conserved among other New World arenaviruses, indicating a common basis of receptor interaction. Our findings also open avenues for the rational development of viral entry inhibitors.

## Introduction

Arenaviruses have single-stranded, bisegmented ambisense RNA genomes and form enveloped virions [1]. Seven arenaviruses cause viral hemorrhagic fever in humans: the Old World arenaviruses Lassa and ‘Lujo,’ and the New World Clade B arenaviruses Machupo (MACV), Junín (JUNV), Guanarito (GTOV), Sabiá (SABV), and Chapare (CHPV) [2], [3], [4]. All of these viruses are US Select Agents and Risk Group 4 Pathogens [5], [6]. MACV, JUNV, and GTOV are also classified as US National Institute of Allergy and Infectious Disease Category A Priority Pathogens [7]. MACV causes human disease outbreak with high case-fatality rates. To date, at least 1,200 cases with ≈200 fatalities have been recorded [8], [9].

Arenaviral genomes encode at least four proteins from two segments (Large and Small). The Large segment encodes a matrix protein (Z) and an RNA-dependent RNA polymerase (L); the Small segment encodes a nucleoprotein (NP) and the glycoprotein precursor GPC [2]. GPC is cleaved by a cellular protease to a stable signal peptide (SSP), and two subunits, GP1 and GP2. The three cleavage products form a stable complex and mediate virus attachment to host cells and fusion of the arenavirion envelope with that of the cell [10], [11], [12], [13], [14], [15]. GP1 mediates the binding of the virion to a cell-surface receptor, whereas the class I fusion protein GP2 mediates membrane fusion after internalization of the virion into an acidified endosome [16], [17]. Interrupting the interaction of GP1 with its cell-surface receptor is a current focus of antiviral development since it would prevent the first and pivotal step of arenavirus host-cell infection.

Transferrin receptor 1 (TfR1) is the principle cell-surface receptor of MACV, JUNV, GTOV, and SABV [17], [18], and a major determinant of host adaptation. However, studies on receptor use and cellular tropism suggest that the non-pathogenic Clade B viruses Amapari (AMPV) and Tacaribe (TCRV) can enter human cells in a human TfR1-independent manner [18], [19], [20]. Nevertheless, AMPV and TCRV use TfR1 orthologs of their principal host animals to infect nonhuman cells [21]. These studies reveal a complex pattern of receptor use for New World arenaviruses, suggesting the existence of additional receptor molecules, and a possible relationship between receptor use and disease potential.

**Figure 1:**
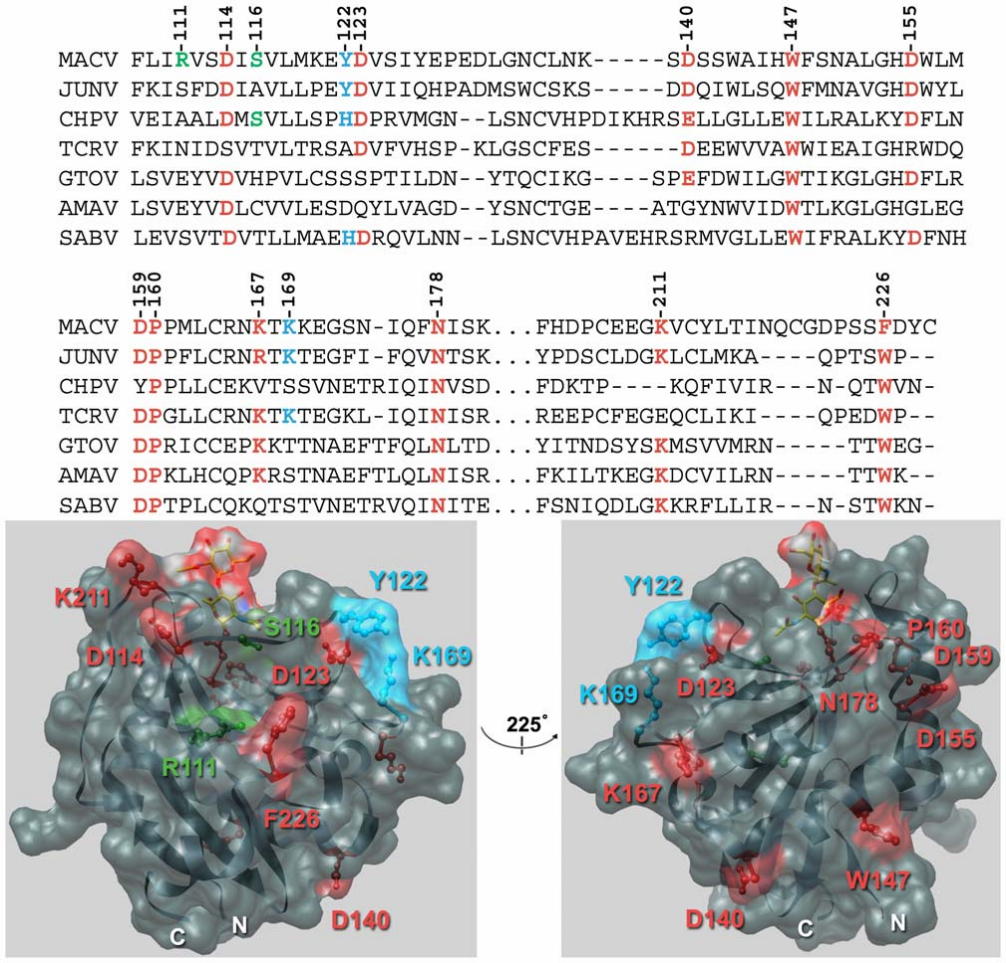
Machupo virus GP1 mutants. Top panel, sequence alignment of New World clade B arenaviruses. Sequence alignment of Machupo virus (MACV) GP1 residues 108–229 with Junín virus (JUNV), Chapare virus (CHPV), Tacaribe virus (TCRV), Guanarito virus (GTOV), Amapari virus (AMAV) and Sabiá virus (SABV) GP1. Chosen residues for mutational analyses are indicated in bold red (conserved), bold blue (partially conserved), and bold green (nonconserved). GenBank Accession numbers for reference sequences are: AMAV, BeAn 70563 (AF512834); CHPV, 810419 (EU260463); GTOV, INH-95551 (AF485258); JUNV, MC2 (D10072); MACV, Carvallo (AY619643); SABV, SPH114202 (U41071); TCRV, (M20304). Bottom panel, structure of MACV GP1 fragment 87–239 [6], orientated with the N- and C-termini to the bottom (PDB ID number: 2WFO).

The GP1-binding site on human TfR1 (hTfR1) has been pinpointed to a prominent loop within the apical domain (residues 201–212) [22]. Conversely, until recently the TfR1-binding site on arenavirus GP1 has been poorly characterized. One study showed that the 20 N-terminal amino acids of MACV GP1 are dispensable for TfR1-binding as a MACV GP1 variant lacking these residues (MACV GP1Δ, residues 79–258) binds hTfR1 even more efficiently than wild-type GP1 [17]. A second study demonstrated that the central region of GTOV GP1 (residues 85–221) interacts with hTfR1 and that residues 159–221 are essential for this interaction [23]. A recently published crystal structure of a truncated MACV GP1 (region 87–239) [24], as well as a co-crystal structure of the MACV GP1∶TfR1 complex [25] provided structural insight of GP1 residues that contact TfR1. The latter study identified five interaction motifs in the interface between MACV GP1 and the apical domain of TfR1 involving some relatively conserved GP1 residues, such as D114 (motif 2), D123 (motif 4), and F226 (motif 3) (colored red, Fig. 1), some less conserved residues, such as Y122 (motif 4) and K169 (motif 4) (colored blue, Fig. 1), as well as some nonconserved residues, such as R111 (motif 1),and S116 (motif 2) (colored green, Fig. 1) [25]. To evaluate experimentally the functional significance of these residues and other conserved solvent-accessible MACV GP1 residues in TfR1-mediated entry, we created a panel of MACV GP1 variants replacing the mentioned amino acids with alanine, and tested the variants' ability to bind hTfR1 and mediate MACV entry in simian and human cells experimental systems.

## Results

We aligned the GP1 sequences of MACV (residues 59–258) with those of JUNV, GTOV, and SABV to identify conserved residues mapping to the surface of the determined MACV GP1 crystal structure (DNAStar Lasergene Software). We identified ten residues of interest that did not contact hTfR1 in the co-crystal structure yet are conserved in at least three of these viruses: D140, W147, D155, D159, P160, K167, N178, and K211 (colored red, Fig. 1).

**Figure 2:**
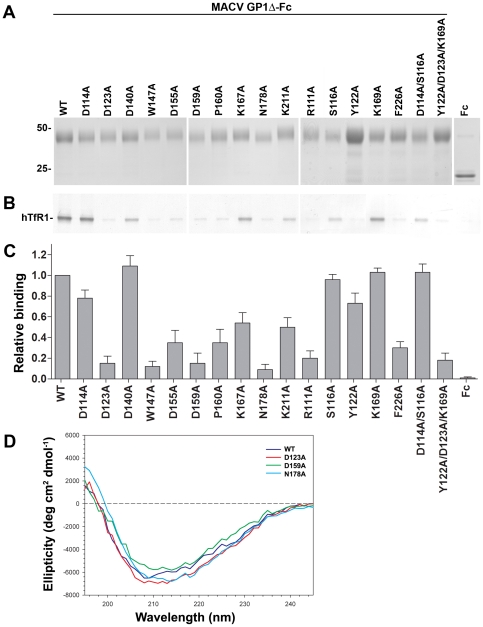
Binding of MACV GP1Δ variants to hTfR1 and the surface of MACV-susceptible cells. A, expression of wild-type MACV GP1Δ, mutants thereof, and Fc control. The numbers to the left of the blots indicate relative molecular mass in kDa. B, ability of wild-type MACV GP1Δ and variants thereof to co-immunoprecipitate hTfR1. Shown is a representative western blot from two independent experiments. C, ability of these proteins to bind to the surface of MACV-permissive (Vero) cells as analyzed by flow cytometry. This assay was performed twice in duplicates yielding similar results. Bars indicate average of duplicates in one representative experiment. Results were normalized by subtracting measurements with secondary antibody only. D, far-UV circular dichroism (CD) of wt GP1Δ and mutants D123A, D159A, and N178A.

We followed a strategy previously employed for virus receptor identification to evaluate the importance for TfR1 binding of these conserved residues, as well as GP1 residues recently indicated in the published MACV GP1∶TfR1 crystal structure [17], [26]. Briefly, we designed codon-optimized open reading frames (ORFs) of MACV GP1Δ in which one or more of the chosen residues were mutated to alanine, and generated them by gene synthesis (DNA2.0). These ORFs were cloned into a pCDM8-like expression vector [17], [26] with the signal sequence of human CD5 upstream and the Fc region of human immunoglobulin G1 downstream of the ORF. Fc alone and wild-type MACV GP1Δ-Fc, which was described previously [17], served as controls. Proteins were purified as described [17], [26]: the individual plasmids were transfected into HEK 293T/17 cells (ATCC) using the calcium-phosphate method and grown in 293 SFM II medium (GIBCO-Invitrogen). Media were harvested and clarified, and Fc fusion proteins precipitated with protein A-sepharose Fast Flow beads (GE Healthcare). Fc fusion proteins were eluted with 3 M MgCl_2_, dialyzed in PBS, and concentrated. Purified proteins were assayed by SDS-PAGE followed by Bio-Safe Coomassie staining (BIO-RAD), and measured using the Quant-iT Protein Assay kit (Invitrogen). All mutant plasmids expressed proteins (Fig. 2A). We evaluated the ability of the individual mutants to bind to hTfR1 using co-immunoprecipitation (co-IP) assays as described [17], [22], [26]. Briefly, HEK 293T/17 cells were transfected with plasmid encoding hTfR1 [17], [22] and lysed 48 h post transfection with RIPA Lysis and Extraction buffer (Thermo Scientific Pierce). Cleared lysates were added to equimolar amounts (200 nM) of Fc, MACV GP1Δ, or variants thereof, and bound proteins were immunoprecipitated with protein A-sepharose Fast Flow beads. hTfR1 in the cell lysate immunoprecipitates was analyzed by SDS-PAGE and western blot using the WesternBreeze Chromogenic kit (Invitrogen) and a murine monoclonal anti-TfR1 antibody (Invitrogen).

Four mutants, D114A, D140A, K167A, and K169A, precipitated hTfR1 as efficiently as wild-type (wt) MACV GP1Δ or at slightly lower levels. Reduction in GP1 interaction with hTfR1 was observed with mutants S116A, K211A, and D114A/S116A (motif 2 in [1]), whereas mutants R111A, Y122A, D123A, W147A, D155A, D159A, P160A, N178A, F226A, and the triple mutant Y122A/D123A/K169A effectively inhibited MACV receptor interactions (Fig. 2B). We performed cell-binding assays as described [17], [26] to determine the ability of these mutants to bind to the surface of MACV-permissive cells. Briefly, Fc constructs were added to 5×10^5^ Vero cells (ATCC) to a final concentration of 200 nM. Cells with bound proteins were incubated with a 1∶40 dilution of FITC-conjugated anti-human Fc antibody (Sigma-Aldrich). Cell-surface binding of constructs was detected by flow cytometry. Baseline fluorescence was determined by measuring cells treated only with the secondary antibody, which was then subtracted from binding values of the tested constructs and control proteins. Overall, cell-binding of mutants supported the co-IP results as mutants that precipitated hTfR1 exhibited high cell-surface binding and vice versa (Fig. 2C). Exceptions were Y122, S116A and D114A/S116A (motif 2), which bound the cell-surface of Vero cells as or almost as efficiently as wt MACV GP1Δ, yet exhibited lower hTfR1 levels in the co-IP assays. The K167A mutant exhibited an opposite trend, as it bound less efficiently to the cell-surface than wt MACV GP1Δ whereas it immunoprecipitated hTfR1 as effectively.

We next tested whether gross misfolding was the reason for the inability of some of the mutants to precipitate hTfR1 and to bind to the cell-surface of MACV-permissive Vero cells. We performed far-UV circular dichroism (CD) of the wt GP1Δ and selected mutants (Fig. 2D). Far-UV CD spectra were taken in PBS at 5°C with a Jasco-810 spectropolarimeter and a 1 mm pathlength rectangular quartz cell. Protein solutions used were from 0.05 to 0.08 mg/ml. Ten scans were accumulated and averaged, without smoothing. The spectra all revealed a single broad minimum in the range of 108 to 216 nm. These results are consistent with similar folding of the wt and mutant proteins, although small changes in the conformation of the mutants cannot be ruled out (the Fc portion of the protein as well as GP1Δ contributed to the measured signal).

**Figure 3:**
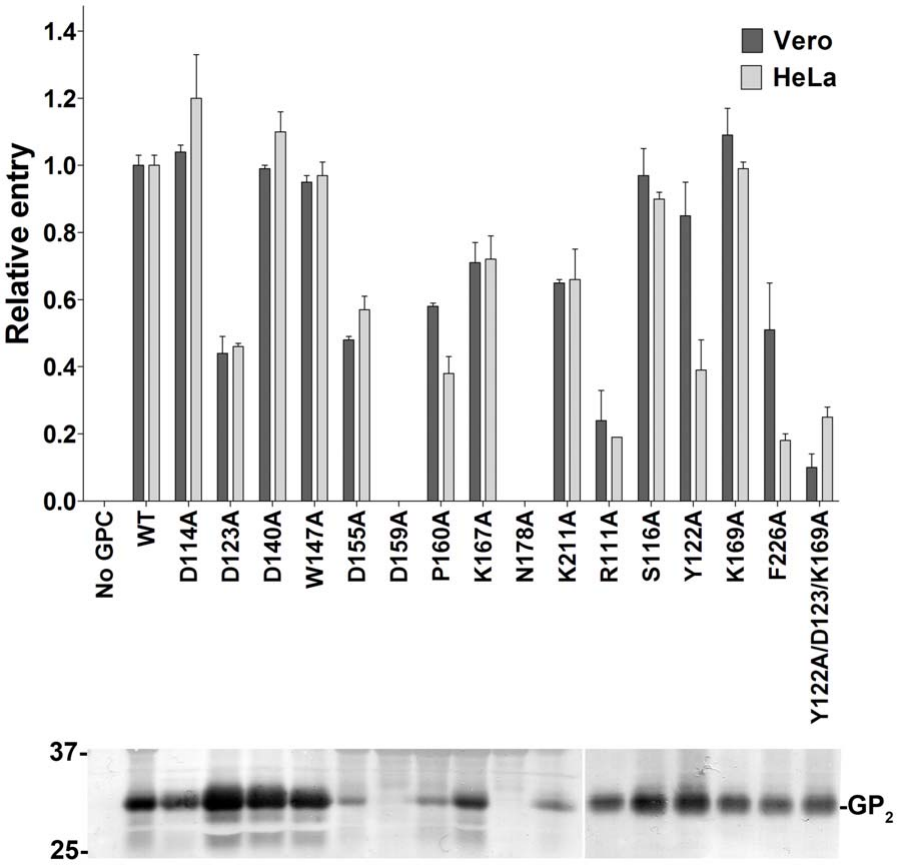
Cell transduction efficiency of eGFP-expressing retrovirus pseudotyped with wild-type MACV GPC, or variants thereof (grey bars, HeLa cells; black bars, Vero cells). This assay was performed twice in triplicates yielding similar results. Bars indicate average of triplicates in one representative experiment. A representative western blot analysis of the various MoMLV pseudotypes is shown using an antibody against MACV GP2. The numbers to the left of the blots indicate relative molecular mass in kDa.

We then cloned ORFs encoding mutant GP1s into plasmids expressing full-length MACV GPC. Retroviral pseudotypes were created as described [17], [26]: HEK 293T/17 cells were transfected by the calcium-phosphate method with plasmid encoding 1) MACV GPC and variants thereof, together with 2) the pQCXIX vector (Clonetech) expressing enhanced green fluorescent protein (eGFP) flanked by the Moloney murine leukemia virus (MoMLV) long terminal repeats, and 3) plasmid containing the MoMLV *gag/pol* genes. Cell supernatants were cleared and measured using the Retro-X™ qRT-PCR Titration kit (Clonetech). Equivalent amounts of pseudotypes were adsorbed on MACV-permissive cells for 2 h. After 48 h, cells were stained with Hoechst 33342 (Invitrogen) and the percentage of eGFP-positive cells was determined by high-content quantitative image-based analysis using an Opera confocal imager (Perkin Elmer). Cells exposed to mock pseudotypes (media of cells transfected with the pQCXIX vector and the MLV *gag/pol* plasmid) were used as negative controls. Western blot analysis of MACV GP2 expression in the producer cells (data not shown) and in the various MoMLV pseudotype preparations (Fig. 3 lower panel) was performed using an antibody against MACV GP2 (New England Peptide) to examine expression, processing, and incorporation levels of wt MACV GPC and variants thereof. In agreement with the co-IP and cell-binding data, MoMLV carrying GPC mutants D114A, S116A, D140A, and K169A had no effect on entry into human (HeLa) and nonhuman primate (Vero) cells (Fig. 3). Mutants D123A, D155A, P160A, F226A, K167A, and K211A had an intermediate effect on MACV entry, with the first four amino acids playing a more prominent role. Interestingly, MACV GPC mutant F226A had a much more pronounced effect on HeLa cell entry (>80% reduction) than on Vero cell entry (>40% reduction).

D155A, P160A, and K211A mutants also exhibited decreased incorporation levels into MoMLV pseudotypes. However, as the cell-entry assay results are in agreement with our results using purified proteins (Fig. 2B and 2C), we suggest that the observed phenotype is not due to decreased incorporation levels but due to the mutations themselves. MACV GPC mutants N178A and D159A were not incorporated into MoMLV pseudotypes and therefore were unable to transduce either cell line. MACV GPC mutant W147A behaved unexpectedly in that it had no effect on MoMLV transduction efficiency, despite its decreased precipitation of hTfR1 and attachment to the cell surface of MACV-permissive cells (Fig. 2). We hypothesize that W147A might be misfolded in the context of MACV GP1Δ but folded correctly in the context of full- length GPC. The two mutants with the most significant effect on cell entry were the R111A mutant and the triple mutant Y122A/D123A/K169A, exhibiting more than 70% reduction in MoMLV transduction efficiency when compared to wt GPC. Finally, the Y122A mutant, while minimally affecting entry into Vero cells, decreased MoMLV entry into HeLa cells. This is in agreement with our assays using purified proteins (Fig. 2), in which the Y122A mutant bound efficiently to the cell surface of Vero cells (Fig. 2C) yet was unable to immunoprecipitate hTfR1.

## Discussion

**Figure 4:**
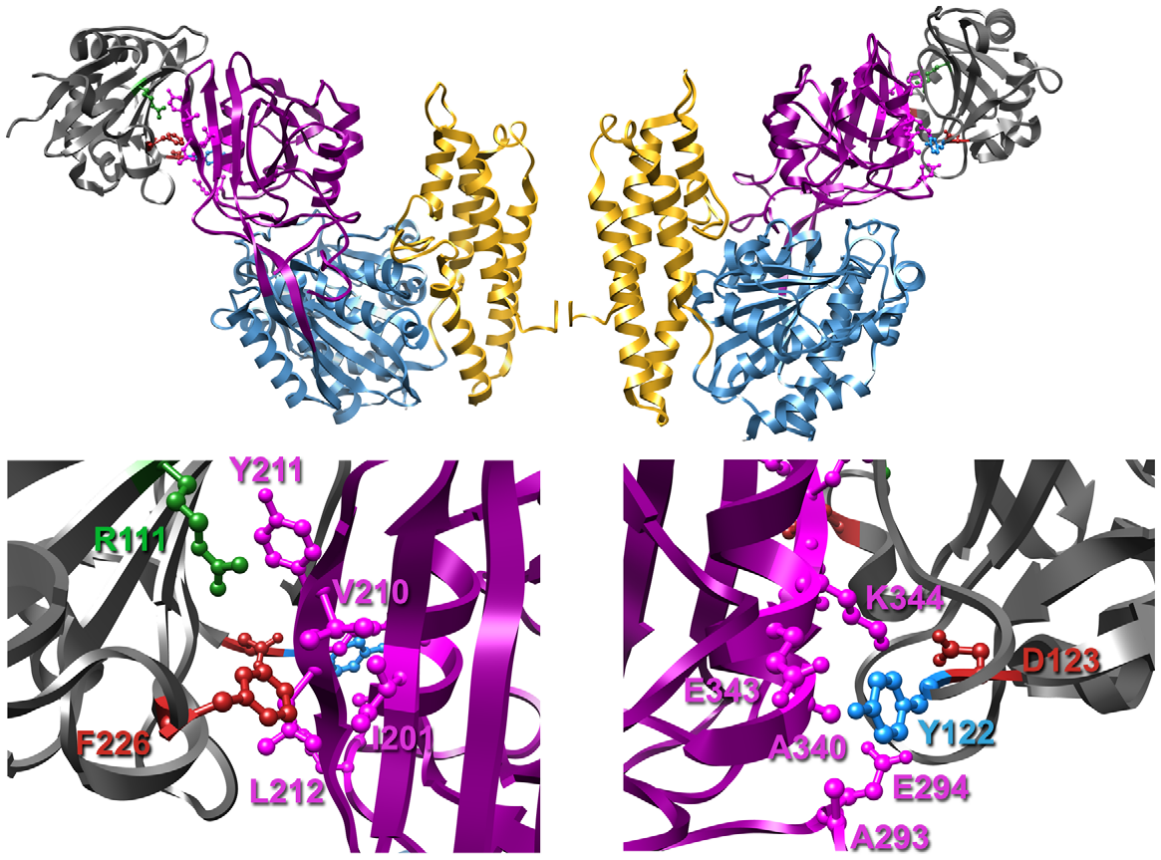
MACV glycoprotein determinants for hTFR1 binding and cell entry. Top panel, structure of the MACV GP1-hTfR1 complex (as described in [25]) with the cell surface orientated to the bottom (PDB ID number: 3KAS). The TfR1 protease-like, helical, and apical domains are colored blue, gold, and magenta, respectively. MACV GP1 is colored in dark grey. Mutated residues are colored as in Fig. 1. Bottom panel, enlargement of the TfR1∶MACV GP1 contact sites. MACV residues important for TfR1 binding are labeled and colored as in Fig. 1. TfR1 residues are labeled and colored in magenta.

In summary, our results demonstrate that GP1 residues R111, D123, Y122, and F226 are important for hTfR1 binding and cell-entry of MACV (Figs. 2 and 3). These results are in accordance with a recent study demonstrating that the central region of GTOV GP1 (residues 85–221) interacts with hTfR1 and that residues 159–221 are essential for this interaction [17]. These results are also in agreement with the recently published structure of MACV GP1 bound to hTfR1 [25]. MACV GP1 R111, which is not conserved in any of the other New World arenaviruses, is part of interaction motif 1 and makes prominent contacts with hTfR1 βII-2 strand, namely V210 (Fig. 1, top panel, and Fig. 4). F226, unique to MACV (W in other New World arenaviruses), is part of interaction motif 3 and has a hydrophobic interaction with hTfR1 V210, as well as a van der Waals contact with hTfR1 I201 and L212 (Fig. 1, top panel, and Fig. 4). D123 and Y122 are part of interaction motif 4 and are conserved between MACV, JUNV, and SABV. D123 forms a hydrogen bond with hTfR1 K344, whereas Y122 forms one with hTfR1 E343 and in addition contacts hTfR1 residues A293, E294, and A340 (Fig. 1, top panel, and Fig. 4). The MACV GP1 Y122A mutant, while minimally affecting entry into Vero cells, decreased MACV MoMLV entry into HeLa cells (Fig. 3). In accordance, this mutant bound efficiently to the cell surface of Vero cells (Fig. 2C) yet was unable to immunoprecipitate hTfR1 (Fig. 2B). These results suggest that Y122 might be important for binding hTfR1 but dispensable for binding simian TfR1, or that additional receptors/attachment factors are facilitating binding and entry of MACV into Vero cells. Whereas TfR1 residues E343, A293, and A340 are conserved among human and simian TfR1, glutamate 294 is an aspartate in simian TfR1 and the proximal asparagine 292 is a lysine.

MACV GPC residues D114, S116, D140, K169, and most likely W147 are dispensable for cell attachment and entry of MACV. According to the crystal structure, D114 and S116 form interaction motif 2, which contacts hTfR1 residues N348, S370, and K371. K169, a part of interaction motif 4, contacts K344, and E294 of hTfR1. In our functional assays, using both purified proteins and MoMLV pseudotypes, mutating these residues had no or minimal effect on hTfR1 binding and cell-entry (Figs. 2 and 3), suggesting that although they might contact hTfR1, they by themselves play no significant role in hTfR1 usage by MACV.

Residues D155, P160, and K211 of MACV GPC had an intermediate effect on hTfR1 binding and cell-entry, with the first two amino acids being more predominant. These residues were not found to contact hTfR1 residues in the crystal structure and could exert their effect on cell-surface binding and entry by affecting overall or local folding of MACV GP1 and possibly by interacting with a yet unknown attachment or entry factor. P160 is conserved among all Clade B New World arenaviruses (Fig. 1, top panel), which use TfR1 as a receptor [1], [21]. However, D155 is conserved only among Clade B New World arenaviruses that cause hemorrhagic fevers in humans and bind hTfR1. Amapari (AMAV) and Tacaribe (TCRV) viruses, which are considered non-pathogenic for humans [2] and which do not bind hTfR1 but bind TfR1 of their mammalian hosts [21], do not have an aspartate residue in this position (Fig. 1, top panel).

Finally, it is difficult to establish the role of MACV GPC residues N178 and D159 in cell binding and entry of the virus. In the context of purified GP1Δ, mutation of these amino acids did not affect overall folding (Fig. 2D), but in the context of GPC these mutants failed to be incorporated into MoMLV pseudotypes. These results suggest that mutagenesis of N178 and D159 might lead to small changes in folding of GP1 (not being detected by CD) that might affect accessibility of hTfR1-binding residues in their vicinity. Alternatively, trafficking, folding, or binding of GP1 to SSP or GP2 might be defective only in the context of full- length GPC. The MACV GP1-hTfR1 crystal structure revealed seven *N*-linked glycans on Asn178 [25]. It is plausible that these glycans play an important role in MACV glycoprotein folding, trafficking, GP complex formation, or entry. N178 and D159 (with the exception of CHPV) are conserved among all Clade B New World arenaviruses (Fig. 1, top panel), which use TfR1 as a receptor [1], [21].

We have used purified MACV GP1 and MoMLV pseudotyped with the MACV glycoproteins in all experiments. Several studies suggest that retroviral vectors pseudotyped with the glycoproteins of arenaviruses adopt the receptor-binding characteristics of the corresponding viruses [17], [20], [27]. Therefore, we believe this surrogate system to be valuable for initial studies of viral cell binding and entry. However, it is important that the system is simplistic and uses only a few viral proteins in the absence of other viral-encoded proteins or genomic elements. Therefore, any results obtained with this system should be verified with infectious virus. Unfortunately, no reverse genetics system has been developed for MACV to date, making it impossible to evaluate the effect of the mutated residues on entry of infectious MACV.

Currently there is no FDA-approved treatment to stop the spread of MACV once humans are infected. Defining interactions between the viral glycoprotein and a cellular receptor may allow for drug development that could limit viral spread in the infected individual and reduce infection during an outbreak. Our mutagenesis analysis of MACV GP1 experimentally elucidates the structure and functional interaction motifs of MACV GP1 and its receptor hTfR1. These studies therefore provide a solid platform for the future design and development of small molecule inhibitors and antivirals specifically targeting viral entry.
